# Experiences and needs of persons who have undergone limb amputation in Saki West, Oyo State, Nigeria

**DOI:** 10.1093/inthealth/ihae068

**Published:** 2024-11-13

**Authors:** Elizabeth Oluwamayowa Oloruntola, Chioma J Eze, Gloria O Alao, Mercy Opateye, Oluwaseun T Gbadebo, Precious E Akinbote, Ruth D Adesina, Oluwadamilare Akingbade

**Affiliations:** College of Nursing Sciences, Baptist Medical Centre, Saki, Oyo State, Nigeria; Faculty of Nursing, University of Ibadan, Oyo State, Nigeria; Institute of Nursing Research, Osogbo, Osun State, Nigeria; Institute of Nursing Research, Osogbo, Osun State, Nigeria; Faculty of Nursing, University of Alberta, Edmonton, AB, Canada; Institute of Nursing Research, Osogbo, Osun State, Nigeria; Institute of Nursing Research, Osogbo, Osun State, Nigeria; School of Nursing, Babcock University, Ilishan-Remo, Ogun State, Nigeria; Oyo State School of Basic Midwifery, Kishi, Oyo State, Nigeria; Institute of Nursing Research, Osogbo, Osun State, Nigeria; Institute of Nursing Research, Osogbo, Osun State, Nigeria; Department of Nursing Services, LAUTECH Teaching Hospital, Osogbo, Osun State, Nigeria; Institute of Nursing Research, Osogbo, Osun State, Nigeria; Faculty of Nursing, University of Alberta, Edmonton, AB, Canada; Faculty of Nursing, Chrisland University, Abeokuta, Ogun State, Nigeria

**Keywords:** amputation, challenges, coping strategies, experiences, limbs, needs

## Abstract

**Background:**

Amputation is a life-changing experience involving the surgical removal of a body part. However, little is known about the experiences of persons who have undergone limb amputation in the Saki West Local Government Area (LGA), which prompted this study.

**Methods:**

An exploratory qualitative study design was used. Thirty participants were interviewed using face-to-face focus group discussions. Five sessions were conducted, with six participants in each session. Data were analysed using the thematic analysis framework of Braun and Clarke.

**Results:**

A total of 76.3% of the participants were males and 73.3% were married. Their ages ranged from 22 and 69 y, with a mean age of 48.0 y and a monthly income of 20 000 naira (US$12). Three major themes emerged from the study: the needs and challenges of those who have undergone amputation, coping strategies of those who have undergone amputation and recommendations by persons who have undergone amputation. The study revealed that people who have undergone amputation experienced psychological pain, such as a loss of self-worth and stigmatization from friends and loved ones. Coping strategies identified were accepting the situation and emotional support from family. Participants recommended providing financial support, empowerment programs, employment opportunities and fostering a community of amputees in the Saki West LGA.

**Conclusions:**

Amputees undergo psychological and psychosocial problems that can affect their health and recovery. They need emotional and financial support, rehabilitation services and provision of prostheses from family, society and the government. These services should be adequately provided in the Saki West LGA, the largest among the 10 suburban local governments in Oyo State, with numerous commercial activities and a high risk for road traffic accidents.

## Introduction

An amputation is an action taken to surgically remove a part of the human body following trauma, disease or congenital conditions.^1^ Surgically, amputation of extremities is classified into major and minor. Major limb amputation is defined as the severance of an extremity proximal to the wrist or ankle.^2^ Globally, 65 million people live with limb amputation, with 1.5 million undergoing amputations, mostly lower limbs, each year.^3^ A study conducted at the Federal Medical Centre (FMC), Birnin Kebbi, Nigeria, in June 2015–May 2016 revealed that 47 amputations were carried out in 47 patients: guillotine amputation was done initially in 8 patients while 39 patients had a definitive primary amputation, 22 patients had a below-knee amputation, 12 patients had an above-knee amputation and other types included in the study were above-elbow amputation, below-elbow amputation, ray amputation, disarticulation and gritti-strokes.^4^ A retrospective review of surgical records of lower limb amputation performed in the University of Nigeria Teaching Hospital revealed that 93 amputations were performed between January 2013 and December 2017.^2^ Also, a study carried out between January 2015 and December 2019 in the Enugu State University of Technology Teaching Hospital showed 22 amputations involving 13 males and 9 females; amputation of the lower limb occurred in 15 cases, while the upper limb was amputated in 7 cases.^5^ These previous studies reveal that Nigeria has an increasing number of amputees in its population.

In the northern and eastern parts of Nigeria, trauma and malignant tumours are the leading indications for extremity amputation.^4^ The common foot complications of diabetes mellitus, peripheral arterial occlusive disease, neuropathy leading to tropical ulcers and soft tissue and/or bone sepsis are related directly to the observed increasing incidence of lower extremity amputation worldwide.^6^ Previous studies conducted in FMC Birnin Kebbi in 2019 and the University of Maiduguri Teaching Hospital in 2019 showed that the most common indication for amputation was traditional bone setter's gangrene (44.7%), followed by diabetes foot gangrene (25.5%), trauma (10.7%), malignancies (6.4%), camel bite (6.4%), peripheral vascular disease (2.1%), burns (2.1%) and damn nuisance limb (2.1%). Diabetes mellitus neuropathy was the leading indication for amputation and below-knee amputation constituted a majority of the amputations.^4,7^

Amputees are faced with several difficulties, including the overt social stigma associated with limb loss, compromised ability to bear weight and the inequality of leg lengths, which are mandatory for maximal and satisfactory function in any individual. The quality of life of the amputee becomes suboptimal, even with acceptable prosthetic rehabilitation.^8^ Amputees are not only stressed due to the loss of a body part, but also due to social role limitations and the need to adjust to changed lifestyle options.^9^ The culture, family and social network of an individual influence the experience and needs of the amputees. Depression is a psychological response among amputees that may linger for years and negatively impact their attempt to adjust socially and psychologically to their physical environment.^10^ The physical disability resulting from amputation causes amputees to feel disabled and incapacitated, resulting in emotional vulnerability and existential uncertainty. Job insecurity, social stigma and changes in family roles were some of the experiences highlighted by persons who have undergone amputation in a previous study carried out between September 2018 and January 2019 at a primary care setting in Singapore.^11^

Amputation is one of the major causes of poor functioning, disrupted daily living activities and socio-economic challenges.^12^ Amputees need a balanced and healthy environment to express their emotions; the extent of physical and emotional pain and symptoms can also be influenced by cultural and religious traditions.^13^ A study on the assessment of psychological well-being, social support and coping strategies of patients with amputation , conducted in Lagos, Nigeria in 2021, revealed that most of the respondents in the study had positive coping strategies, such as believing in prayers, moving closer to God and engaging in spiritual activities, using social support, not distancing themselves from people around them, browsing the internet excessively to engage their mind and watching TV alone. Only a few respondents consume alcoholic drinks and use excessive drugs to cope with psychological distress.^14^

People with disabilities or dysfunctions experience challenging situations when navigating their environment,^15^ which can adversely affect their health and recovery. Amputees described experiences of stigmatization and relegation, which negatively impact relationships and employability; there were also limited rehabilitation services, and those available were unaffordable to the masses.^16^ Delayed wound healing and an inability to acquire prostheses due to financial constraints have also been reported among this population; hence, lower limb amputees were trained and mobilized using axillary crutches, which were some of the challenges faced by amputees.^5^ However, little is known about the experiences of persons who have undergone limb amputation in the Saki West Local Government Area (LGA), which prompted this study. This study aims to answer the following research question: What are the experiences and challenges faced by individuals who have undergone limb amputation in the Saki West LGA? By exploring these experiences, the study provides essential insights into the psychological, social and practical challenges faced by amputees, which are crucial for developing targeted interventions and support systems. The findings will inform healthcare providers, policymakers and support organizations, enabling them to design and implement more effective care plans and health education programs tailored to the needs of amputees. Thus the study not only enriches the academic understanding of limb amputation, but also has practical implications for improving the quality of care and support available to this population.

## Methods

### Study design

An exploratory qualitative study was conducted among individuals in the Saki West LGA who have had a limb amputated.

### Research setting

The Saki West LGA is one of the 33 LGAs of Oyo State, Nigeria, having its administrative headquarters in Saki town. It is the largest local government among the 10 suburban local governments (Oke-Ogun) in Oyo State. Saki town is situated in the northern part of Oyo State, western Nigeria. Saki is known as ‘the food basket of the state’ because of its agricultural activities. Similarly, it is home to a large number of commercial activities. Hence there is a large percentage of motorcycle and vehicular transportation within and outside the town, which predisposes people to road traffic accidents for reasons such as bad roads, inadequate training for drivers and riders and refusal to use safety precautions.

### Study population and sampling strategy

A purposive sampling technique was used to select 30 participants of different ages (22–69 y), tribes and socio-economic backgrounds. This was done to capture the experiences of a wider group of participants represented in the LGA. As the purposive sampling technique is prone to selection bias, the selection criteria were clearly defined *ab initio* and strictly adhered to. Similarly, we ensured reflexivity during the selection process, whereby the research team members reflected on their individual biases during the selection process.

Face-to-face focus group discussions (FGDs) were conducted. FGDs were considered suitable as they allow for in-depth insights and rich qualitative data and give real-time feedback and instant reactions. This is because the facilitator, who takes the lead role, asks open-ended questions to elicit detailed information.^17^ Five FGDs were conducted until data saturation was reached. The inclusion criteria were individuals who had undergone a form of limb amputation in any hospital in Nigeria, could communicate fluently in English or Yoruba and willingly consented to participate in the study. Exclusion criteria were amputees who are minors and those with cognitive impairment.

### Data collection

Two of the authors, female registered nurses with bachelor's degrees, conducted the interviews and were the facilitators. Four other research assistants are student nurses. They were trained by the project supervisor, a PhD holder in nursing and experienced in qualitative data collection. An information sheet including detailed information about the study was provided to all the participants and was read by the research assistants; this was also explained by the facilitators, after which participants were required to sign a written consent form. Demographic data were collected with a demographic data sheet.

As evidence suggests that focus group participants should be homogeneous concerning at least one characteristic, our participants for each focus group were homogeneous regarding age, social and economic backgrounds. This allowed for free and lively discussions in each focus group.

The interview was conducted in the Saki West local government secretariat conference hall. Some measures were taken to improve the quality of the data collection. The conference hall was free of noise and distractions and the assistants presented the ground rules before the data collection. Similarly, we ensured peer debriefing, which involved discussing the data collection process and findings with colleagues or other researchers, which helped provide feedback and identify potential biases. In each session, one of the interviewers moderated the discussion as a facilitator, while the other acted as an observer to handle the logistics of recording the interviews and monitoring the participants’ reactions.^18^ Some of the questions asked included what led to the amputation, how they handled the news when it came, how they have been coping with having a limb amputated and their recommendations.

The FGDs were conducted in both English and Yoruba, depending on the participants’ language preferences to ensure they could fully express their experiences and challenges. To ensure the accuracy and reliability of the data, a translation and back-translation process was rigorously followed. The process involved the following steps: Initially, one of the research team members who is fluent in both English and Yoruba translated the interview guide and consent forms from English to Yoruba. The translations were then reviewed by another bilingual researcher to ensure accuracy and clarity. During the interviews, responses in Yoruba were recorded and later transcribed into English. The English transcripts were then back-translated into Yoruba by a separate translator to verify the consistency and accuracy of the translations. Any discrepancies were resolved through discussions among the research team to ensure that the final data accurately represented the participants’ original responses.

The interview lasted an average of 45 min–1 h per session. Some measures were taken to ensure rigor during data collection and data analysis. Open-ended questions were utilized to ensure credibility. We ensured dependability and transferability through audit trails. Similarly, the first and eighth authors reviewed participants’ quotes and interpretations to confirm congruence and validate the findings.^18,19^

### Data analysis

Data were analysed using the QSR NVivo 12 software program (Lumivero, Denver, CO, USA). Verbatim transcription was conducted while listening to the recording. The thematic analysis method prescribed by Braun and Clarke^20^ was used for data analysis to identify themes and patterns in the data. This method has six phases: familiarization with the data (this is the first stage of thematic analysis and it involves taking a broad look at the data and examining it as a whole), generating initial code (involves methodically coding the data's notable characteristics and applying labels that explain what the data entail), searching for themes (involves compiling several codes into a theme), reviewing themes (to confirm that the themes are generated appropriately and accurately represent the data), defining and naming the themes (involves giving names and definitions to each theme) and producing the report (involves putting the findings into writing).^20^ The Consolidated Criteria for Reporting Qualitative Studies guidelines were adhered to in the reporting of this study.

## Results

A total of 30 participants were interviewed. The participants were 22–69 y of age, with a mean age of 48.0 y. A total of 76.3% were male, 73.3% were married, 60% were Muslim and 86.7% lived with family members. Two-thirds (66.7%) were artisans and 66.7% had amputation of a body part after a traumatic incident. A total of 80% had their amputation done in a hospital in Nigeria and 83.3% earned a monthly income of 20 000 naira (NGN; US$12) (see Table 1).

**Table 1.** Sociodemographic data of the participants

		Frequency	
Variable		(n=30)	Percent
Age (years)	20–29	2	6.7
	30–39	7	23.3
	40–49	8	26.7
	50–59	7	23.3
	60–69	6	20.0
	Mean (SD) 48±(17.31)		
Gender	Male	23	76.3
	Female	7	23.3
Marital status	Single	5	16.7
	Married	22	73.3
	Separated	1	3.3
	Widowed	1	3.3
	Divorced	1	3.3
Ethnicity	Yoruba	30	100.0
	Igbo	–	–
	Hausa	–	–
	Others	–	
Religion	Christian	12	40.0
	Islam	18	60.0
	Other	–	–
Education	None	1	3.3
	Primary	11	36.7
	Secondary	9	30.0
	Tertiary	9	30.0
Who are you living with?	Family members	26	86.7
	Alone	4	13.3
Reason for limb amputation	Trauma	20	66.7
	Disease	7	23.3
	Perceived unnatural cause	2	6.7
	Mismanagement	–	–
	Birth defects	1	3.3
Where was the amputation done?	Hospital outside Nigeria Hospital in Nigeria In utero Non-hospital in Nigeria	3 24 1 2	10.0 80.0 3.3 6.7
Monthly household income, NGN (US$)	<20 000 (43)	25	83.3
	21 000–50 000 (46–110)	4	13.3
	51 000–100 000 (113–217)	1	3.3
	101 000–150 000 (220–326)	–	–

Close to one-third (30%) were traders, accounting for 30% of the respondents, 20% were retired, 10% of the respondents were civil servants and 10% were unemployed, while administrators and fashion designers each accounted for 7%.

### Themes and subthemes

Three major themes emerged: needs and challenges of people who have undergone amputation, coping strategies of those have undergone amputation and recommendations by those who have undergone amputation. These themes were further elaborated into various subthemes (see Table 2).

**Table 2.** Themes and subthemes generated from the interviews

Themes	Subthemes
Experiences and challenges of people who have undergone amputation	Pain and traumaDemoralising nature of the eventStigmatization from friends and loved ones
Coping strategies of those who have undergone amputation	Acceptance of the situationEmotional support from family
Recommendations from persons who have undergone limb amputation	Build and foster a community of amputeesProvision of financial supportProvision of empowerment programs and job opportunities for disabled people

### Theme 1: Experience and challenges of people who have undergone amputation

Participants described the experience of amputation as extraordinarily challenging, encompassing a range of profound emotional and psychological difficulties. This theme explores the multifaceted nature of their experiences, including the significant pain and trauma they endured, the demoralizing impact on their daily lives and sense of self and the stigmatization they faced from those around them. Their narratives reveal a deep struggle with the physical and emotional aftermath of losing a body part, including enduring pain, grappling with altered life circumstances and confronting social rejection. These experiences are further elucidated through the following subthemes:

#### Pain and trauma

Participants described undergoing amputation and adjusting to the loss of a body part as a deeply painful and traumatic experience. The profound physical and emotional suffering was evident, with accounts highlighting not only the intense pain, but also the significant psychological impact. Participants spoke of their overwhelming sorrow and mental anguish, reflecting on how the experience left them in a state of distress and despair.

Ah! It was painful, and I was very sorrowful all through that phase. (patient 26, 27 y, male, artisan)

 

The whole incidence was traumatic for me. Often, I cried and cried…. (patient 12, 28 y, female, civil servant)

 

I was becoming mentally unstable, and I wasn't happy again; the pain was just too much for me to bear. (patient 13, 32 y, male, trader)

#### The demoralizing nature of the event

Participants experienced the loss of a body part as profoundly demoralizing, with the impact extending beyond physical limitations to affect their overall sense of self and ability to engage in previously familiar activities. Many reported a significant decline in their mental and emotional well-being, describing feelings of hopelessness, loss of appetite and a lack of strength, which led to withdrawal from social and daily activities. This sense of demoralization was compounded by practical challenges, such as difficulties in performing daily tasks, concerns about future life events such as having more children and shifts in career aspirations. The loss of employment and reliance on others for basic needs further exacerbated their sense of despair.

I thought all hope has been dashed. I was indoors, I lost appetite, no strength, I was just like a skeleton. I was completely demoralised and became withdrawn. (patient 22, 59 y, male, trader)

 

The accident happened at the early stage of my life. Carrying out daily activities was very difficult. I just had one child, so I thought I wouldn't be able to give birth to another child. (patient 23, 59 y, female, civil servant)

 

Growing up, I dreamed of becoming a medical doctor. But things did not remain the same again after the accident that led to amputation. I just resorted to learning a trade. (patient 29, 47 y, male, trader)

 

It's not easy. I lost my job after the incident, and I haven't been able to secure a job; I can't readily provide my needs without alms from friends. (patient 28, 35 y, male, artisan)

#### Stigmatization from family and friends

Participants commonly reported experiencing stigmatization from family and friends following their amputation. Many felt rejected and judged by those close to them, which significantly compounded their emotional distress. The stigma was evident in various forms, including negative remarks about their physical abilities and social exclusion. Some participants described how their relationships deteriorated, with friends and family distancing themselves or expressing derogatory views. This social rejection not only deepened the participants’ sense of isolation, but also contributed to their overall psychological distress.

After I got my artificial leg, my friends complained that I always walk slowly. I had many girlfriends, but they all ran away. Some ridiculed me and called me a one-legged man. (patient 29, 47 y, male, trader)

 

Even those that have been my friends for ages suddenly stopped coming to see me, some of my siblings separated from me because they thought a disabled person won't be useful in life again. (patient 24, 56 y, female, trader)

 

I went into depression because family members and friends all ran away because of the responsibilities that came with my condition. (patient 23, 59 y, female, civil servant)

### Theme 2: Coping strategies

Despite the immense challenges and trauma associated with amputation, participants developed various coping strategies to navigate their new realities. The process of adjusting to life after amputation involved several key approaches. Central to these strategies was acceptance of their situation, which many found essential for emotional recovery and adaptation. Additionally, emotional support from family and friends played a crucial role in the coping process. Supportive relationships provided essential reassurance and practical help, mitigating feelings of loneliness and enhancing overall well-being. This is captured under the following subthemes:

#### Acceptance of the situation

Participants emphasized that accepting their amputation was crucial for coping. Many found that coming to terms with their situation allowed them to move forward and find peace, despite initial struggles and counselling efforts. Embracing their condition often led to emotional recovery and the ability to support others in similar situations. In contrast, those who struggled with denial faced prolonged healing, increased complications and greater difficulty adjusting. This highlights the critical role of acceptance in the recovery process and the significant challenges faced by those unable to reconcile with their condition.

…In the long run, people did everything they could to counsel me, but it proved abortive. Until I called myself to order…at least my life was spared; I only lost a body part. (patient 12, 28 y, female, civil servant)

 

Mine is different; I used to wake up most nights and think all hope was lost. But other times, I also wake up and ask myself, what if I had died? These days, whenever I visit other amputees in the hospital, I encourage and inspire them. (patient 11, 33 y, male, unemployed)

 

We resigned to our fate and thanked God for being alive. Today, we have given birth to both male and female children. (patient 24, 56 y, female, trader)

In contrast, those who lived in denial had a prolonged healing time, developed more complications and found it difficult to cope.

I was the only survivor of the accident; when I got to the hospital, the doctor told me that my leg would be amputated, but I felt a tingling sensation in my leg, so I concluded that there was life in the leg. Therefore, I told my parents to take me back home…I was thinking my leg would not be cut…. (patient 4, 55 y, male, trader)

#### Emotional support from family members and friends

Participants highlighted the vital role of emotional support from family and friends in their coping process after amputation. Many found that encouragement and regular visits from loved ones helped prevent feelings of loneliness and depression, significantly boosting their emotional well-being. This support was not only emotional, but also practical, providing financial assistance and care that alleviated the burdens of their condition. Those without such support often faced greater emotional challenges, underscoring the essential role of a strong support network in recovery.

Relatives supported me emotionally by visiting regularly. Their visit helped me not to feel lonely and depressed. (patient 20, 22 y, male, student)

 

…They didn't segregate me and didn't see me as different because I didn't have two legs. It helped my self-confidence because I never felt I could not do anything others with complete limbs did. (patient 13, 32 y, male, trader)

 

You know it is therapeutic when you have family support. It was easy to take the burden and challenges away from my mind because I was not lonely and didn't give myself to thinking. Loneliness can drift your mind to things you shouldn't even think about. (patient 12, 28 y, female, civil servant)

 

…My doctor friend visited, and he encouraged me not to engage in deep thoughts because of my condition. My friends advised me to keep calm and that I would get to wherever I was destined to be. (patient 18, 60 y, male, retired)

 

If not for my family members, I would have died long ago. They provided me financial support, care, love, hospitality and everything; they stood by me. (patient 22, 59 y, male, trader)

 

Hmmmm, my family members were sad, but they helped and supported me financially, ah! I thank God. If not, so ah! The incident would have taken my life. Because the money was so much at last, we thank God I am still alive. (patient 19, 50 y, male, artisan)

 

My sister was there for me, and my stepsiblings stood by me, even at the hospital, when maggot was coming out of the hand, and it was smelling, my siblings did not leave me. (patient 21, 37 y, male, trader)

 

People with this kind of condition need support. So, if they don't have people around them, they might think more than expected to the extent that they may want to kill themselves. But if they have people to support them, the incident might cost their lives. (patient 20, 22 y, male, student)

### Theme 3: Recommendations from persons who have undergone limb amputation

The participants offered several recommendations for supporting people who have undergone amputation. They recommended the provision of financial support, empowerment and job opportunities and building a community of amputees; their ideas are captured in the following subthemes:

#### Build and foster a community of amputees

Participants recommended that creating and nurturing a community of amputees could offer significant benefits. Such a community would provide opportunities for individuals to find inspiration, encouragement and motivation from others who share similar experiences or have successfully navigated similar challenges. One participant noted that the mutual support among amputees in the hospital greatly uplifted their spirits, contrasting sharply with the isolation and difficulties they faced once home.

Just like what you said without lying, I was much happier at the hospital with other amputees than at home in town…We encouraged each other, and I was assured. Once I got home, challenges bumped into my face, making me sad. (patient 5, 50 y, male, artisan)

#### Provision of financial support

Participants emphasized the need for financial assistance from the government, non-governmental organizations (NGOs) and wealthy individuals due to the high costs of hospitalization, surgery and prosthetics. They suggested that such support could ease the financial burden and help with recovery. Recommendations included providing direct financial aid, supporting victims’ families and creating employment opportunities for amputees to alleviate their stress and improve their situation.

Some individuals who are rich can help us with funds to get prostheses. The first prosthesis, I did, I know how much I spent to be able to get it. (patient 13, 32 y, male, trader)

 

What the government should do is support the victims’ family so that the victim will not be having worrisome thoughts more than expected. (patient 17, 65 y, male, retired

 

The cost of the operation and everything has drained me. Just look at me; what kind of job can I still do? I have been at home for the past three years…What can I do with one leg and a half? I think the government and some organisations should organise programs to help people financially. (patient 22, 59 y, male, trader)

 

Ways the government can help are diverse. The government can collect data on amputees, e.g. in Saki, and place them on salary or employ them. Although there are amputees who are financially buoyant…I am not buoyant, I need help, if the government gives me 10 million I would be happy. (patient 11, 33 y, male, unemployed)

#### Provision of empowerment programs and employment opportunities

Participants advocated for the provision of equitable job and empowerment opportunities for disabled individuals. They emphasized that such support could help them integrate into society and achieve financial independence. Recommendations included ensuring fair job access, providing business start-up assistance and creating income-generating opportunities to reduce dependency and improve overall well-being.

What we are saying is that the government should help us; they should help us in this condition we have found ourselves. The government can also give us equal job opportunities with our colleagues who are not disabled. (patient 24, 56 y, female, trader)

 

Yes! The government and private organisations should have mercy on us and empower us. If they can help us get a business, we can live as normal humans as we are. We also want to have a source of income to cater for our needs… (crying). (patient 30, 44 y, male, driver)

 

…you see, if they help us with money to do business, we would eat at the right time. Having unnecessary thoughts would be minimal, unlike when one is not working and looks up to people for what to eat. (patient 7, 60 y, female, trader)

#### Build and foster a community of amputees

Participants recommended fostering a community of amputees to provide mutual support and encouragement. They felt that being among others with similar experiences was comforting and motivating, as it offered reassurance and inspiration. This sense of community was seen as a crucial element in coping with the challenges and emotional difficulties associated with amputation.

Just like what you said without lying, I was much happier at the hospital with other amputees than at home in town. Because we all had similar issues, I was more comfortable in their midst; we encouraged each other, and I was assured. Once I got home, challenges bumped into my face, making me sad. (patient 5, 50 y, male, artisan)

## Discussion

This study investigated the experiences and needs of persons who have undergone limb amputation in Saki West LGA, Oyo State, Nigeria. The first theme revealed the experiences and challenges of people who have undergone amputation. Our results show that people who have undergone amputation in Saki experienced psychosocial problems such as a loss of self-worth and stigmatization from family and loved ones. Statements from the participants in this study showed that a lack of self-worth, pain and stigmatization from friends are some of the challenges faced by amputees in Saki. This is consistent with a study conducted in northern Uganda, where the participants in that study said that stigmatization and bullying are part of the challenges and experiences that they faced following an amputation.^15^ This highlights the need for appropriate interventions to address the psychosocial issues of these patients, and evidence suggests that nurses can help reach patients in underserved areas.^21–23^

Similarly, in a study conducted by Kizilkurt et al.^24^ on the quality of life after lower extremity amputation due to diabetic foot ulcer, it was noted that emotional stress is closely associated with amputation. This is because when a body part is distorted, it causes an inadequate feeling about one's body, leading to low self-esteem, because how the body is perceived has a huge effect on social, physical, mental and overall quality of life. Conversely, a study on the experiences and needs of patients with lower limb amputation in Saudi Arabia showed that participants’ adaptation to body image enabled mental adjustment after undergoing amputation.^12^ It also agrees with the study on the experiences of people with diabetes-related lower limb amputation in the Komfo Anokye Teaching Hospital (KATH) in Ghana, where participants coped with the outcome of undergoing amputation after they consoled themselves.^25^ However, this contrasts with a study on quality of life and functionality after lower limb amputations, which revealed that participants showed less acceptance of their status as amputees, impairing their ability to cope with their condition.^26^ This variation underscores the importance of personalized coping strategies that consider individual differences in adaptation and self-acceptance. The differences in adaptation observed across studies suggest that a one-size-fits-all approach to psychological support may not be effective. Instead, interventions should be tailored to address the specific needs and coping mechanisms of each individual.

The second theme focused on participants’ coping strategies. Family and friends’ support was the participants’ major coping strategy. This agrees with a study where amputees and patients with chronic illnesses described family support as the major source of psychological therapy.^25,27^ The role of the family in supporting patients in Nigeria has been documented. This suggests that when designing interventions to meet the needs of this population, researchers might consider designing family-focused interventions. However, a study conducted at KATH in Ghana, revealed that participants equally considered spirituality as a significant form of coping strategy.^25^ Spiritual well-being in a chronic illness is highly important. The World Health Organization has identified spirituality as one of the health dimensions. An impairment in spiritual well-being causes loneliness, depression and a reduced ability to cope with problems.^28^ Spiritual coping may help to combat symptom burden, leading to an improvement in the quality of life because of the awareness that comes with prayer, meditation and self-reflection, which influences illness perception, leading to adaptation to life events.^29^ Integrating spiritual support into interventions may enhance coping strategies and overall well-being.

The third theme captured the recommendations that the amputees suggested in the study: the provision of monetary support from government and NGOs; the provision of not only jobs, but also empowerment programs; and building and fostering a community of amputees. This is important, as evidence suggests that poverty has remained a significant issue in the country, where >100 million people live on less than US$1 per day. The country is among those with the highest number of people living in acute poverty.^30^ Similarly, this study shows that most participants were low-income, with 83.3% having a <US$22 monthly household income, and around one-third were traders, with 20% retired and 10% unemployed. This suggests that financial support from the government, NGOs and well-to-do individuals to procure prostheses, start a business or take care of their families is highly recommended. Participants in our study also recommended that providing empowerment opportunities and equal job opportunities for amputees will go a long way in handling their financial hardship. This finding agrees with the findings of a study in Uganda where amputees described disability as posing a serious financial threat to individuals and their dependents.^15^ Another study reported that people living with upper limb amputation in Kampala, Uganda need occupational support services.^31^ Financial support and empowerment opportunities are essential for improving the quality of life and economic stability of amputees.

## Implications for Policy Development

The first national health plan in Nigeria was developed in 1947 to provide universal healthcare to the citizens.^32^ In 1993 the Nigerian government announced the disability decree to provide clear and comprehensive legal security and protection for Nigerians with disabilities and to establish enforcement of the rights and privileges under this decree and other laws applicable to those with disabilities in Nigeria. They include but are not limited to Nigerians with Disabilities Decree 1993, disability protection laws in several Nigerian states, the National Policy on Disability in Nigeria 2017 and the Discrimination Against Persons with Disabilities (Prohibition) 2018 Act, which prescribes sanctions such as incarceration or pecuniary forfeit. In addition to these and other national inclusion strategies, some NGOs have made visible efforts towards educating the public on disability and encouraging disabled people that disability does not mean inability.^33^ Some of the major acts, as regards this study, are disabled people must not be discriminated against in any way in public transportation facilities and service providers should make a provision for people with disabilities to have easy access to their services. Public organizations are to offer employment to the disabled equal to 5% of their workforce. Also, people with disabilities can file a lawsuit for damages.

Unfortunately, corruption and misuse of public funds, unavailability of funds, lack of community participation and public enlightenment, constant changes in health policy by the government and a lack of trained personnel are some of the reasons why health policies in Nigeria are not implemented.^34^ Balogun^32^ found that a lack of political will and commitment and poor governance by successive governments, corruption, an underfunded health system, a large informal sector economy and extreme poverty are some of the reasons why healthcare policies are not implemented in Nigeria.

With the experiences shared in this study by people living with disabilities, it was found that the concerns identified in 1993, 2017 and 2018 have not been resolved. Thus we recommend that the Nigerian government should implement and enforce the rights of people living with disabilities in Nigeria and also create a formidable amputee support group to oversee the welfare of the group, provide employment, advocate for the members and ensure the implementation of policies.

## Limitations and Recommendations

This research addressed a significant gap in knowledge by providing unique insights into the experiences of persons with disabilities and will serve as a background for future research. However, as this study was conducted in just one LGA and the number of participants was limited, the findings might not be generalizable to other LGAs throughout the country. Similarly, as the participants were mostly low-income, the findings might not be generalizable to high-income earners who have undergone amputation in Nigeria. Thus these limitations should be considered when interpreting the findings.

It is recommended that other states in Nigeria conduct similar studies to increase the evidence of prosthetic needs so policymakers can develop policies to aid people living with amputated limbs and develop subsidized prosthetic services. Furthermore, studies on coping strategies and perception of self after amputation should be carried out in communities with large numbers of amputees and orthopaedic hospitals. Moreover, it is recommended that those living with a disability should be adequately represented and included in the leadership of government establishments and offered employment in government offices, based on their level of ability and should not be discriminated against. Additionally, it is recommended that research should be conducted among owners of NGOs to determine the extent of training and education given to persons with disabilities to allow them to face the realities of the outside world.

## Conclusions

The lived experiences of people with an amputated limb in Nigeria have been understudied in the literature. The contribution of this study is the documentation of the needs and experiences of people who have undergone limb amputation in Saki West LGA, Oyo State, Nigeria. We found that these experiences are predominantly negative and are comparable to the concerns of the disability rights law of Nigeria, which was signed in 2018. There is a need for concerted efforts to ensure that the needs of this group are well taken care of.

## Data Availability

The study data are available upon reasonable request to the corresponding author.
